# Secure Screw Placement in Management of Acetabular Fractures Using the Suprapectineal Quadrilateral Buttress Plate

**DOI:** 10.1155/2017/8231301

**Published:** 2017-06-15

**Authors:** R. J. Egli, M. J. B. Keel, J. L. Cullmann, J. D. Bastian

**Affiliations:** ^1^University Institute of Diagnostic, Interventional and Paediatric Radiology, University of Bern, Inselspital, Bern, Switzerland; ^2^Department of Orthopaedic and Trauma Surgery, University of Bern, Inselspital, Bern, Switzerland

## Abstract

Acetabular fractures involving predominantly the anterior column associated with a disruption of the quadrilateral surface can be treated with instrumentation implementing the stabilization of the quadrilateral surface. The recently introduced suprapectineal quadrilateral buttress plate is specifically designed to prevent secondary medial subluxation of the femoral head, especially in elderly patients with reduced ability for partial weight bearing. Whereas there are guidelines available for safe screw fixation for the anterior and posterior columns, there might be a concern for intra-articular placement of screws placed through the infrapectineal part of the quadrilateral buttress plate. Within this report we analyzed retrospectively screw placement in 30 plates in postoperative CT scans using algorithms for metal artifact reduction. None of the screws of the buttress plate penetrated the hip joint. We describe the placement, length, and spatial orientation of the screws used for fracture fixation and suggest that the use of intraoperative image intensifiers with a combined inlet-obturator view of 30–45° best projects the screws and the hip joint. Preoperative knowledge of approximate screw placement and information for accurate intraoperative imaging may contribute to safe acetabular fracture fixation and may reduce operating time and limit radiation exposure to the patient and the personnel. This trial is registered with KEK-BE: 266/2014.

## 1. Introduction

**Figure 1:**
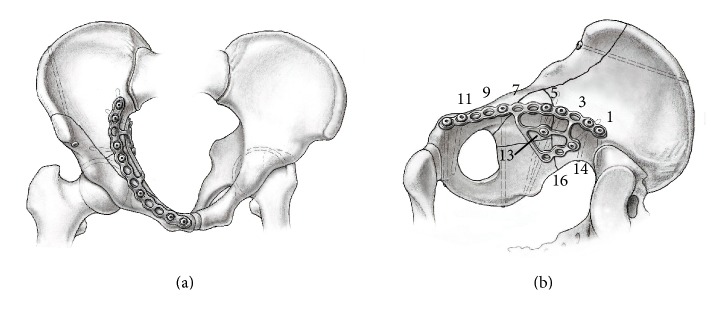
Illustration of a reduced typical acetabular fracture with disruption of the quadrilateral surface and the positioning of the quadrilateral buttress plate in an (a) inlet and (b) intrapelvic view with the numbered screw holes.

Acetabular fractures of the anterior column or wall, frequently with involvement of the posterior column (both column, anterior column with posterior hemitransverse fracture or transverse fractures) and an associated disruption of the quadrilateral surface (Figure 1) with consecutive medial displacement of the femoral head, show a high incidence especially in elderly patients [1–3]. Surgical treatment is most often required as only poor results are achieved with conservative treatment [4–6]. Anatomical restoration, however, remains cumbersome for several reasons: (i) the fracture located in the false and true pelvis impeding visualization and access of the fracture lines; (ii) osteopenia with a poor bone stock and insufficient partial weight bearing of the treated limb in elderly patients raising the risk for failure of the osteosynthesis with secondary loss of reduction; (iii) risk of penetration of the hip joint by screws around the iliopectineal eminence with consecutive development of secondary osteoarthritis. Concerning (i), we recently showed that using the Pararectus approach for surgical exposure and treatment of acetabular fractures significantly improved access in the false pelvis and provides versatility for fracture fixation as compared to the modified Stoppa approach [7, 8]. In particular, the Pararectus approach allowed for a longer posterior column screw to be used and screws could be placed more posteromedially towards the posterior inferior iliac spine or the ischial tuberosity. Concerning (ii), a new plate design was developed and introduced into market, the suprapectineal quadrilateral buttress plate (Stryker Osteosynthesis AG, Selzach, Switzerland) [9] (Figure 1). This design is composed of a plate for suprapectineal fixation of the anterior and posterior columns and a perpendicular quadrilateral buttress plate to stabilize the quadrilateral surface. Previously, plate designs providing quadrilateral surface buttressing proved superior in terms of fracture stability and prevention of medial subluxation as compared to traditional fracture fixation techniques [9]. Concerning (iii), screw placement with respect to “safe zones” preventing penetration of the hip joint and injury to neurovascular structures is well documented for reduction of the anterior and posterior column fractures [10–12] by the intraoperative use of an image intensifier. However, secure placement of the screw for fixation of the quadrilateral buttress plate is challenging due to its anatomical relationship to the acetabulum. Apart from the general guidance of using intraoperative image intensifier with a combined inlet-obturator view for periacetabular screw fixation, no further guidelines are available.

With this report we provide guidance for screw fixation of the quadrilateral buttress plate by giving details of screw lengths used and their spatial orientation after fracture reduction. Moreover we provide angles for inlet and obturator view to be used during intraoperative imaging for optimized projection of the spatial orientation of the screws and the hip joint. These data will on the one hand ascertain extra-articular screw placement and on the other hand may lead to optimized intraoperative imaging potentially reducing radiation exposure to the patient and the personnel. To that end, we retrospectively analyzed the positioning of the osteosynthesis in postoperative computed tomography from 30 plates used in 29 patients.

## 2. Material and Methods

### 2.1. Patients

Between September 2014 and March 2016, 31 patients were treated for acetabular fractures with the suprapectineal quadrilateral buttress plate. Two patients (two plates) were excluded, because no screw fixation of the quadrilateral buttress plate was carried as a stable fixation through the holes of the suprapectineal part of the plate was already achieved. The surgeries were performed by the coauthor MJBK, the head of trauma surgery.

### 2.2. Approach and Instrumentation

Surgical access was achieved using the Pararectus approach as described previously [8, 13, 14] with an incision length of approximately 12 cm. The screws for fixation of the suprapectineal plates (screws 1–12, Figure 1) were positioned according to the safe zones for reduction of anterior and posterior column fractures [10–12]. Depending on the fracture anatomy, holes 4–8 were used as posterior column screws and holes 7–10 as infra-acetabular screws [7]. The positioning of the screws of the quadrilateral buttress plate (screws 13–16) was guided by an image intensifier (Siremobil, Siemens Medical Solutions, Zurich, Switzerland) with a combined inlet-obturator view (C-arm tilted 25° cranially and rotated 15° towards the injured side) as proposed for periacetabular screw fixation.

### 2.3. Postoperative Computed Tomography (CT) Scan

Each patient received a CT scan of the pelvis a few days after surgery as a standard of care in our institution to ascertain appropriate reduction of acetabular fractures and to assess retrospectively the positioning of the osteosynthesis. CT scans were performed on a Somatom Definition Flash or Somatom Definition Edge 128-Slice (Siemens, Forchheim, Germany). Tube voltage was 100–140 kV. The patients were placed in the supine position on the CT table for native CT scans. All scans were performed using the automatic dose modulation software (CARE Dose mAs, Siemens, Forchheim, Germany). Collimation was 0.6 mm. Image reconstructions were performed with a slice thickness of 0.6 mm in increments of 0.3 mm using the soft tissue kernel (I30 with iterative strength 3) and bone kernel (B70 with extended CT-scale). Two methods of metal artifact reductions were applied, either dual energy [15] or iterative metal artifact reduction (iMAR), an algorithm developed by Siemens [16, 17]. These two methods lead to significant improvement of image quality and allow for detailed measurements in close proximity to metal implants.

### 2.4. Position and Fixation of Plate and Screws

The positioning of the plate is mostly guided by the anatomy of the pelvis and the configuration of the fracture. As reference, the distance of the medial and lateral border of the suprapectineal plate to the symphysis and to the iliosacral joint, respectively, were measured. In the suprapectineal plate 12 holes and in the quadrilateral plate four holes are available for screw fixation (Figure 1(b)).

### 2.5. Measurements

**Figure 2:**
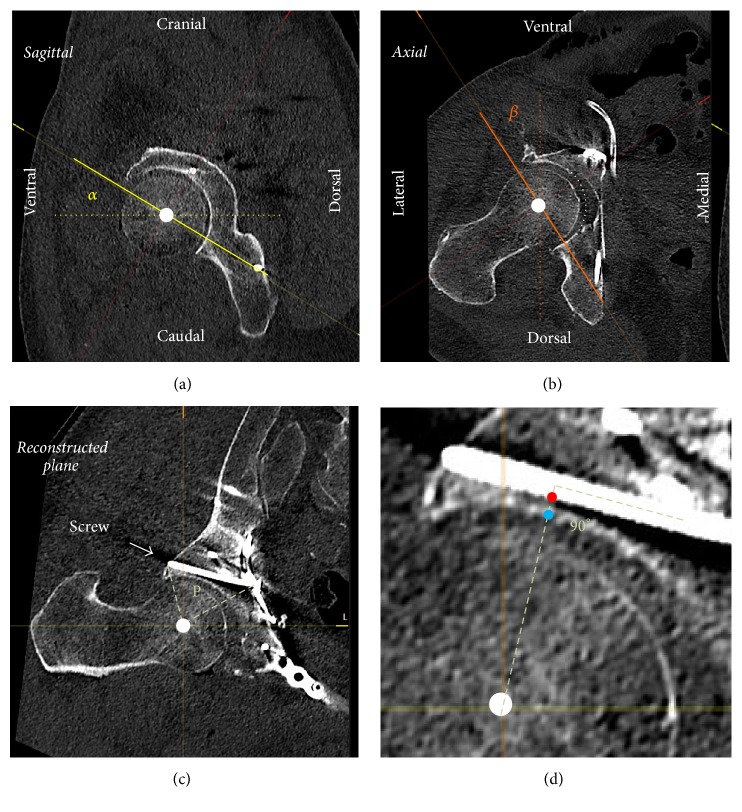
MPR of an acetabular fracture treated with a quadrilateral buttress plate. (a) A combination of inlet view (rotation by the angle *α* around the frontal axis (dotted line) in the sagittal plane) and (b) obturator view (rotation by the angle *β* around the sagittal axis (dotted line) in the axial plane) reconstructs (c) the plane P defined by the center of the femoral head (white dot) and the screw to be analyzed. This plane defines the real spatial orientation of the screw and the hip joint. (d) Detailed view of (c), the screw distance to the subchondral bone of the acetabulum (red to blue dot) was measured on a vertical line to the screw axis through the center of the femoral head. In (b), the white dotted line represents the virtual continuous acetabular subchondral bone in the lunate fossa.

The distance of screws 4–10 and 13–16 to the acetabular subchondral bone was measured in multiplanar reformation (MPR) using PACS software. Therefore, a virtual plane was reconstructed through the length axis of the screw to be analyzed and the center of the ipsilateral femoral head (Figure 2). Within this plane, the distance of the screw to the acetabular subchondral bone was measured perpendicular to the screw axis (Figure 2). One exception is infra-acetabular screws, which travel through or adjacent to lunate fossa if there is no subchondral bone; in this case a virtual continuous subchondral bone was drawn (see Figure 2(b), white dotted line) and the distance to the screw was measured.

Intraoperatively, the placement of the screws of the quadrilateral buttress plate was guided using an image intensifier with a combined inlet-obturator view (see above). However, the optimal angles projecting the real distance of the screws to the joint intraoperatively are essentially only estimated through trial and error. In the MPR of the postoperative CT scan the virtual plane defined by the axis of the screw and the center of the femoral head (Figure 2(c)) was constructed with the femoral head as rotation center. This plane projects the real spatial orientation of the screw and the hip joint. The degrees of rotation around the frontal axis (*α*, Figure 2(a)) and around the sagittal axis (*β*, Figure 2(b)) to construct this plane correspond to the inlet and obturator view angles, respectively, which would accurately project intraoperatively the real spatial orientation of the screw and the hip joint. Malrotation of the pelvis around the longitudinal axis in the CT scan was corrected by the angle between the pubococcygeal line and the sagittal axis of the CT scan. Pelvic inclination was not corrected since it can be assumed that the inclination of a patient in supine position is equal on the CT table as on the operating table.

**Figure 3:**
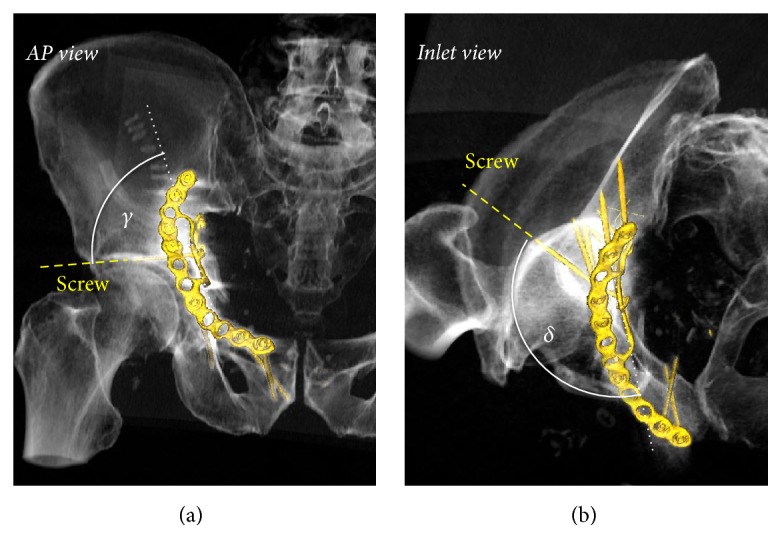
Angulation of screws of the quadrilateral buttress plate. 3D reconstruction of a treated acetabular fracture. (a) In frontal view, the angle between the quadrilateral buttress plate (dotted white line) and the screw defines the craniocaudal angle *γ*. (b) In inlet view the angle between the quadrilateral buttress plate and the screw defines the anteroposterior angle *δ*.

The angulation of screws 13–16 was measured to provide more information on screw placement. The reference plane was defined in MPR by hole 13, hole 16, and the anterior corner of quadrilateral buttress plate (the corner between holes 13 and 16, see Figure 1(b)). Afterwards the angles of the screws craniocaudally in AP view and anteroposteriorly in inlet view were determined in relation to the projection of the reference plane (Figure 3).

## 3. Statistics

For descriptive statistics, either box plots depicting median, quartiles, minimum value, and maximum values or simple mean ± standard deviations were used. The calculations were done with Microsoft Excel V14 (Microsoft Office Professional Plus 2010).

## 4. Results

In 29 patients (22 males aged 62.2 ± 16.9 years, 7 females aged 80.1 ± 14.8 years) 30 suprapectineal quadrilateral buttress plates were implanted for the reduction of acetabular fractures involving disruption of the quadrilateral surface.

### 4.1. Plate Positioning

The distance of the medial border of the suprapectineal plate to the symphysis was 10 mm (median) ranging from 0 to 25 mm. The distance of the lateral border to the iliosacral joint was 0 mm (median) ranging from −16 mm (thus crossing the ISG) to 10 mm.

### 4.2. Suprapectineal Screws

**Figure 4:**
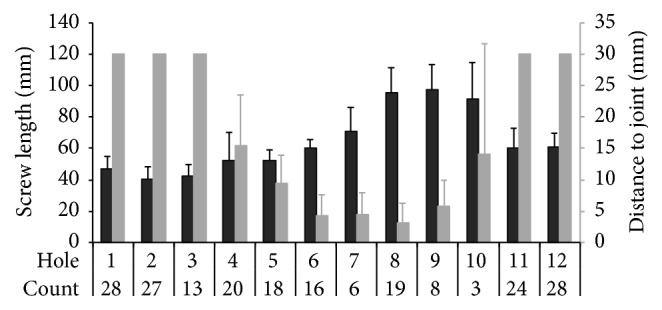
Screw length used for the suprapectineal plate and the distance to the subchondral bone of the acetabulum. Screws in holes 1–3 and 11-12 were always more than 30 mm apart from the acetabulum and were not measured. Count: how often a hole was occupied by a screw (total of 32 plates).

Screw holes used for fixation of the suprapectineal part of the plate and the length of the respective screws are detailed in Figure 4. The screws with the closest distance to the joint space were screws 5–8 with a mean distance of 9, 4, 5, and 3 mm, respectively. In one case, screw number 7 penetrated the subchondral bone of the acetabulum by 1 mm.

### 4.3. Screws of the Quadrilateral Buttress Plate

Four screw holes (13–16) are available for fixation of the quadrilateral buttress plate; however, hole 15 was only used twice; hole 16 was never occupied. Depending on the fracture anatomy and the surgical accessibility, hole 13 only (in 5 plates), hole 14 only (in 7 plates), or holes 13 and 14 (in 18 plates) were used for fracture fixation.

**Figure 5:**
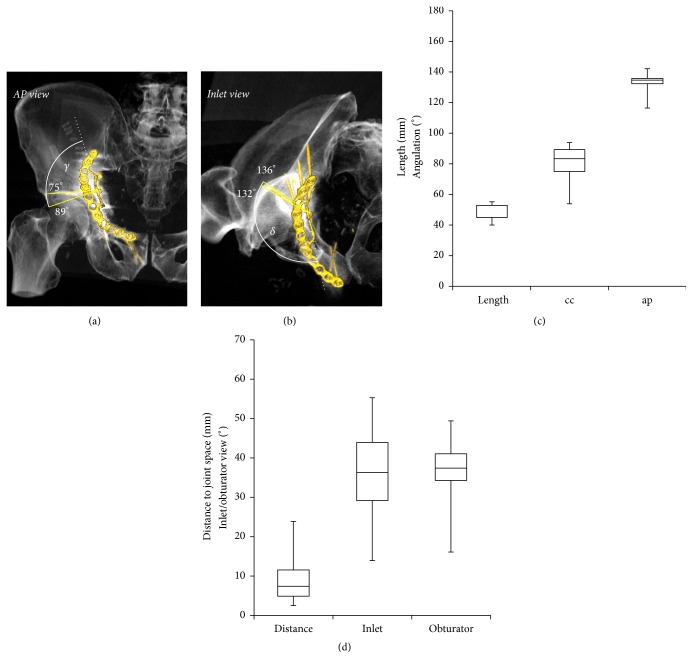
Characterization of screw 13 of the quadrilateral buttress plate. (a) Craniocaudal angles illustrating the range between the 25th and 75th percentile. (b) Anteroposterior angles illustrating the range between the 25th and 75th percentile. (c) Length of the screw, craniocaudal angle (cc), and anteroposterior angle (ap). (d) Distance of the screw to the joint and inlet and obturator view angles projecting the real spatial orientation of the screw an the hip joint. Box plots illustrate minimum, 25th percentile, 75th percentile, and maximum values.

The median length of screw 13 was 45 mm (min–max: 40 to 55 mm) and the median angulations relative to the plane defined by the quadrilateral buttress plate were 83° (54–94°) craniocaudally and 135° (116–142°) anteroposteriorly. The median distance of the screw to the joint space was 7 mm (3 to 24 mm). The inlet and obturator view angles theoretically depicting the real distance were 36° (14–55°) and 38° (16–50°), respectively (Figure 5).

**Figure 6:**
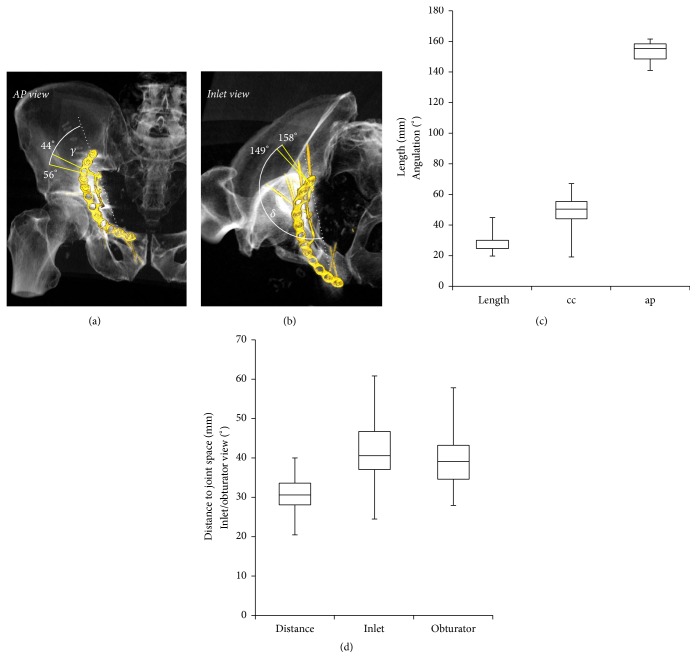
Characterization of screw 14 of the quadrilateral buttress plate. (a) Craniocaudal angles illustrating the range between the 25th and 75th percentile. (b) Anteroposterior angles illustrating the range between the 25th and 75th. (c) Length of the screw, craniocaudal angle (cc), and anteroposterior angle (ap). (d) Distance of the screw to the joint and inlet and obturator view angles projecting the real spatial orientation of the screw an the hip joint. Box plots illustrate minimum, 25th percentile, 75th percentile, and maximum values.

The median length of screw 14 was 30 mm (min–max: 20 to 45 mm) and the median angulations relative to the plane defined by the quadrilateral buttress plate were 51° (19–67°) craniocaudal and 155° (41–61°) anteroposterior. The median distance of the screw to the joint space was 31 mm (20 to 39 mm). The inlet and obturator view angles depicting the real distance were 41° (24–61°) and 39° (28–51°), respectively (Figure 6).

## 5. Discussion

Recently a new osteosynthesis material with a quadrilateral buttress plate was brought to market with the intention to improve fracture reduction and fixation as well as prevent medial subluxation of acetabular fracture with disruption of the quadrilateral surface. We used the generally recommended combined inlet-obturator view on an image intensifier intraoperatively to guide secure screw fixation of the quadrilateral plate. Intraoperative 3D imaging may come along with some advantages compared to 2D imaging in intraarticular fracture reduction [18]; however 3D imaging entails a substantial logistic effort and is not generally available in all institutions. Through analysis of postoperative CT scans and applying algorithms for metal artifact reduction, we showed that none of the screws used for fixation of the buttress plate penetrated the hip joint with a minimal distance of 2.5 mm for screw 13 and 20.3 mm for screw 14. Using intraoperative image intensifier with the beam centered on the femoral head and a combined inlet-obturator view angles around 30°–45° for inlet and 35–40° for obturator for screw 13 and 35°–45° for inlet and 35–45° for obturator for screw 14 allows in most cases for a close to real projection of the screw distance to the joint. This gives the surgeon the most accurate representation of the fracture reduction, which is of particular importance since fracture lines are often close to the hip joint. Importantly, due to projection geometry, the distance estimated intraoperatively cannot be shorter than the real distance; thus a screw in extra-articular projection in the image intensifier always signifies that the joint is not penetrated.

Generally, screw fixation of the suprapectineal plate was performed according to the safe zones for fixation of anterior and posterior column fractures [10–12]. We measured the distance of the suprapectineal screws to the joint space and found once that a screw number 7 was overreaching the subchondral bone of the acetabulum by less than 1 mm. However, this particular screw was travelling through the lunate fossa and in fact did not penetrate the subchondral bone; rather it crossed the line of the virtual continuous acetabular socket as detailed in Figure 2(b). Twelve months after surgery no signs of secondary osteoarthritis were seen.

## 6. Conclusion

Routine CT scans after complex acetabular fractures to ascertain correct fracture reduction allowed for retrospective analysis of plate localization and screw placement of the suprapectineal quadrilateral buttress plate. In contrast to what was expected, the screws placed in the buttress plate were not in danger for intraarticular placement. However, periacetabular screws in the suprapectineal plate appear to be at risk for articular penetration. Further development or refining existing safety tunnels for those screws (not restricted to this particular plate design) may need to be discussed.
